# Behavioral Differences in the Preference for Hepatitis B Virus Vaccination: A Discrete Choice Experiment

**DOI:** 10.3390/vaccines8030527

**Published:** 2020-09-14

**Authors:** Na Guo, Jian Wang, Stephen Nicholas, Elizabeth Maitland, Dawei Zhu

**Affiliations:** 1 China Population and Development Research Center, Beijing 100081, China; guonasun@126.com; 2 Dong Fureng Institute of Economic and Social Development, Wuhan University, Wuhan 430072, China; wangjian993@whu.edu.cn; 3 Center for Health Economics and Management in School of Economics and Management, Wuhan University, Wuhan 430072, China; 4 Australian National Institute of Management and Commerce, Sydney, NSW 2015, Australia; stephen.nicholas@newcastle.edu.au; 5 Research Institute for International Strategies, Guangdong University of Foreign Studies, Guangzhou 510420, China; 6 School of Economics and School of Management, Tianjin Normal University, Tianjin 300074, China; 7 Newcastle Business School, University of Newcastle, Newcastle, NSW 2015, Australia; 8 Management School, University of Liverpool, Liverpool L697ZH, UK; E.Maitland@liverpool.ac.uk; 9 China Center for Health Development Studies, Peking University, Beijing 100191, China

**Keywords:** behavioral economics, preference for vaccination, choice behavior, vaccination, Hepatitis B

## Abstract

Understanding behavioral factors differences in the preferences for vaccinations can improve predictions of vaccine uptake rates and identify effective policy interventions to increase the demand for vaccinations. In this study, 353 adults in Shandong province in China were interviewed about their preferences for hepatitis B virus (HBV) vaccination. A discrete choice experiment (DCE) was employed to analyze the preference for HBV vaccinations, and a mixed logit model was used to estimate respondent preferences for vaccination attributes included in the DCE. While the protection rate against hepatitis B (HB), duration of protection, risk of side-effects, and vaccination cost were shown to influence adults’ preferences for HBV vaccination, adults valued “99% hepatitis B protection” above other attributes, followed by “20 years’ protection duration” and “1 in 150,000 risk of side-effects”. Individuals with lower time discount rates, non-overconfidence, or higher risk aversion were more likely to choose a vaccine. Lower risk aversion individuals showed a higher preference for lower risk of side-effects. Lower time discount rate individuals showed a higher preference for longer protection duration. Non-overconfidence individuals showed a higher preference for higher hepatitis B protection and cost. Interventions should be targeted to the behavioral determinants impeding vaccination.

## 1. Introduction

Vaccination is one of the most important public health interventions, saving 2–3 million lives every year, hundreds of millions of lives in the 20th century, and eradicating diseases such as smallpox [1]. Even though the beneficial impacts of vaccination on society and the individual are well proved, almost every country struggles with “vaccine hesitancy”. Some individuals refuse or delay vaccinations despite the availability of vaccines [2]. The consequences of vaccine hesitancy are serious both to the individual, who gets sick, and the society, as the disease spreads widely. Vaccine hesitancy has resulted in a series of repeated outbreaks of vaccine preventable diseases [3,4] such as measles, which made a comeback in the United States after being eradicated for over 19 years, with severe economy-wide consequences [5,6].

Vaccine hesitancy is complex, and standard rational economic models of decision-making display some weaknesses in explaining and predicting the vaccination decision [7,8,9]. The decision to vaccinate is based on its benefits and costs, which involves various uncertainties, such as the infection risk, vaccine effectiveness, and vaccine side-effects. Some of these uncertainties, such as side-effects, have a very low probability, which makes vaccine hesitancy difficult to comprehend [10]. One problem is that the financial costs of vaccinations occur around the time of the vaccination, while the benefits are reaped at some uncertain future point in time. This means that an individual’s attitude to risk and time, as well as confidence around the vaccine, play important roles in assessing the costs and benefits of vaccinations [11,12,13].

Recent research in behavioral economics, which incorporates insights from psychology, neuroscience, cognition and institutional economics, provides new insights into the vaccination decision [10,14,15,16,17,18]. The cost of the vaccine, risk aversion, time discount rate (or time preference), and overconfidence (or complacency) affect the decision to vaccinate. Risk aversion, which means an individual’s fear of being infected and the vaccine’s side-effects, has a positive effect on the decision to vaccinate [15,16]. Individuals’ time discount (time preference) rates suggest that people with smaller time discount rates (future-oriented or higher time preference individuals) display a greater probability to vaccinate [12,16]. The overconfidence effect, which is the individual’s tendency to underestimate “real” objective risks, does not have direct effects on vaccination decision, but has indirect effects by means of perception variables, such as the subjective probability of infection, and assessment of the effectiveness and side-effects of the vaccine [16,19]. 

Although previous studies have examined the association between behavioral factors, such as risk aversion, time discount rate, and overconfidence, with the vaccination decision, those studies did not account for consumer preferences. Providing an important tool for policymakers, preferences, or an individual’s valuation, can have a major impact on the willingness to use health care services [20]. It is important to understand the differences in vaccination preferences to establish effective interventions that support or complement public health vaccination policies. Drawing on hepatitis B virus (HBV) vaccination data for Shandong province, we evaluate the association between vaccination decisions and behavioral factors, and investigate how preferences vary across individual-specific behavioral factors. 

HBV infection is a major public health issue in China, with an estimated 90 million chronic hepatitis B virus carriers, which accounts for almost 7% of the Chinese population [21]. In 2018, an estimated 1.28 million people were infected by viral hepatitis (dominated by type B) in China, with viral hepatitis the top infectious disease and the third killer infection in China in 2018 [22]. Free vaccination for children from 2002, with catch-up vaccinations for those born between 1995 and 2002, in 2009, dramatically reduced the HBV prevalence in children, but left those in the 15–44 age group displaying a high incidence of HBV [23,24]. These high incident groups born before 1995 must pay for the HB vaccine [25]. However, in 2020, hepatitis imposed a heavy economic burden on HBV sufferers, their families and the society [26]. In Shandong province, Lu et al. estimated the inpatient medical costs of hepatitis B-related diseases ranged from $US2954 to $US10,635 per patient and Zhang et al. found that in Jiangsu province the direct economic costs of HBV-related diseases ranged from $US107 for outpatients to $US3193 for inpatients [27,28]. These prevalence rates and public health costs call for expanded vaccinations of susceptible adults and the need for public health officials to intervene to reduce hepatitis B infections in adults. 

Given the individual and economic costs of HBV in China, understanding behavioral factors differences in the preferences for vaccinations can throw light on vaccine uptake rates and guide policy makers in setting interventions to increase the demand for hepatitis B vaccinations. Equally important, our results from HBV provide insights into the vaccination decision in China generally.

## 2. Materials and Methods 

### 2.1. Sampling and Participants

The HBV vaccination program for adults was used to elicit vaccination preferences and to examine how preferences vary across different individual behavioral factor characteristics. Ten communities in Linyi city Shandong province were chosen on the basis of GDP per capita using a stratified random sampling method to conduct the face-to-face survey. In 2018, 50 individuals in each community were surveyed randomly using a questionnaire. Our response rate was 353 or 70.6%. Free HBV vaccinations for individuals born after 1994, limited adults in our study to those 24 or older.

A detailed explanatory statement of the study was provided to all respondents, which defined the study aims and confirmed no identifiable personal data would be collected and participation was voluntary. Verbal consent of respondents was obtained. The project was approved by the Ethics Committee at Shandong University (No. 201001052).

### 2.2. Study Design

Discrete choice experiment (DCE) methodology is a quantitative research method for eliciting preferences over hypothetical alternative scenarios, including the preferences of adults for different HBV vaccine characteristics that influence their decision to vaccinate [29,30,31]. Displayed in Table 1, the HBV vaccination attributes, comprising protection rates, out-of-pocket (OOP) expenses, protection duration and side-effect risks [32], and their levels were based on the extant literature and a panel of experts [25]. The 108 hypothetical alternatives from combining the four attributes, with each attribute level, meant a single individual was unable to answer all these alternatives, so a sample of alternatives was generated by using an orthogonal experimental design. Thirty-six choice questions addressed all main effects sufficiently, and these questions were divided randomly into 6 different versions of the questionnaire. Each version of the survey included 6 choice questions, with each question describing 2 alternative vaccines in terms of 4 attributes and a “neither vaccine or not vaccinate” option. Participants were asked, “If you were actually offered the 2 vaccines, which would you prefer to choose?” The questionnaire set out a detailed description of the attributes and also collected data on age, sex, level of education, income, health status, perceived risk of HBV infection, perceived severity of HBV, risk aversion, time discount rate, and overconfidence. According to Johnson and Orme [33], the minimum sample size for DCE is 167, which is below our sample of 353 responses.

### 2.3. Behavioral Factors

Follow previous research, three simple questions were used to measure risk aversion, time discount rate, and overconfidence [12,13,15,16]. Risk aversion was measured by: When you usually go out, how high does the probability between zero and 100 percent of rain have to be before you take an umbrella? The group with lower risk aversion was identified by using a cutoff of 50 percentage to generate a binary lower/high risk aversion variable. Time preference was measured by: “In planning your/your family’s saving and spending, which of the following time periods is more important to you and your husband/wife/partner?: next few weeks; next few months; next year; next few years; next 5–10 years; or longer than 10 years”. Participants who chose next year or longer were classified with a low time discount rate. The variable measuring overconfidence of respondents was based on the responses to the statement “I will never be robbed”, with response options 1, strongly agree to 5, strongly disagree. Participants with a score of above 2 were classified as non-overconfidence. 

### 2.4. Control Variables

Information on participants’ sociodemographic characteristics, perceived risk of HBV infection, and perceived severity of HBV. Sociodemographic variables consisted of sex (female or male), age (under 40 or 40 and above), education attainment (primary school or below and secondary school or higher) and income (≤RMB 2000 or RMB > 2000 per month) were collected. Health status was measured by “Would you say your health is very good, good, fair, poor or very poor?”, which were dichotomized into good (very good or good) and poor (fair, poor, or very poor) health. The perceived risk of HBV infection was assigned values by the respondents depending on the answer to the question of “how likely would you be infected by HBV (Percentages between 0 and 100)”. The group with lower risk were identified by using a cutoff of 30 percentage to generate a binary perceived risk of HBV infection variable (0 = Low, 1 = High). The perceived severity of HBV was assigned values depending on how serious (not serious, moderately serious, or very serious) the respondent believed HBV.

### 2.5. Statistical Analyses

Descriptive statistics were used to analyze the non-DCE variables. This study used a mixed logit model, with 500 Halton draws with normally distributed parameters, to estimate participants’ preferences for the HBV vaccine [34]. The mixed logit model allows for attribute coefficients to be randomly distributed, and improves the fit of the model significantly over conditional logit modelling. Each estimated coefficient is a preference weight and represents the relative contribution of the attribute level to the utility that respondents assign to an alternative [35]. There are 3 alternatives in each of the stated choice tasks in the DCE survey: vaccine A, vaccine B, or “neither vaccine or not vaccinate”. The association between individuals’ vaccination decisions to choose “neither vaccine or not vaccinate” and behavioral factors were tested using the mixed logit model with individual behavioral characteristics interacted with the ‘‘neither vaccine or not vaccinate” indicator [25]. For the interacted terms, positive (negative) estimators are correlated with decreased (increased) stated uptake. This study employed a set of models which included interaction between individuals’ behavioral characteristics (including risk aversion, time discount rate, and overconfidence) and all attributes of the vaccine to estimate the potential difference in preference between groups of individuals’ behavioral factors [36].

Following previous studies, we also estimated the changes in the probability of individuals choosing a vaccine with specified attributes [32,37]. These results are present in graphs illustrating the marginal effect of varying one attribute level at a time from the base case, holding all other attributes constant. All heterogeneities were considered in the calculation of the mean uptake, which means that the mean uptake is just not the uptake of someone with average coefficient values [25]. The impact of alternative levels of vaccine attributes on individuals’ preferences inform policymakers in determining vaccine strategies. 

A *p*-value of 0.05 was considered statistically significant. All statistical analyses were performed in STATA 15 (Stata Corp, College Station, TX, USA).

## 3. Results

### 3.1. Respondent Characteristics

Table 2 displays the sample’s descriptive statistics. A total of 53.5% of the sample was male, 62.9% had a high education (years of schooling higher than nine years), and most reported their monthly income as more than RMB 2000. Our respondents reported that 56.7% had “good” or ‘‘excellent” health, 52.4% had a relatively lower risk of infection, and 38.2% had a perceived high severity of HBV. In terms of behavioral characteristics, 46.18% reported a lower risk aversion, 51.56% had a lower time discount rate, and 72.24% were non-confident.

### 3.2. General Preference for HBV Vaccine

Table 3 shows the mixed logit model results for preferences. In the base Model 1, all vaccination characteristics except “10 years’ protection duration” proved to influence adults’ preferences for the HBV vaccination (*p* < 0.05). The variable with the greatest magnitude of association with HBV vaccination preference was “99% hepatitis B protection”, followed by “20 years’ protection duration” and “1/150,000 risk of side-effects”. As expected, respondents demonstrated a negative preference for out-of-pocket costs.

Model 2 shows the mixed logit model with individual characteristics interacting with the “neither vaccine” indicator. For the interacting terms, positive (negative) values are associated with decreased (increased) stated uptake. Those who were older and those who perceived a high risk of infection were more likely to choose an HBV vaccine than to choose ‘‘neither vaccine”. Individuals with a lower time discount rate or non-overconfidence degree were more likely to take the vaccine while those with lower risk aversion were less likely to choose a vaccine and selected “neither vaccine” more often.

### 3.3. Behavioral Factors’ Differences in Preference for Vaccine 

Results from three interaction models are presented in Table 4. In Model 3, the non-significant coefficient of the characteristic level “risk of side-effects” indicates that individuals with higher risk aversion did not significantly prefer to choose one of the side-effect levels over the reference level of side-effects. The positive signs for the interaction terms of ‘side-effects’ and lower risk aversion indicate that adults with a low risk aversion showed a significantly higher preference for “lower risk of side-effects” than adults with a high risk aversion. In Model 4, “20 years’ protection duration” was more important to adults with a lower time discount rate compared to adults with a higher time discount rate. Compared to adults with overconfidence, non-overconfidence respondents showed a higher preference for “99% protection against HBV” and willingness to pay more for vaccination (Model 5).

### 3.4. Change in the Probability of Participation 

**Figure 1:**
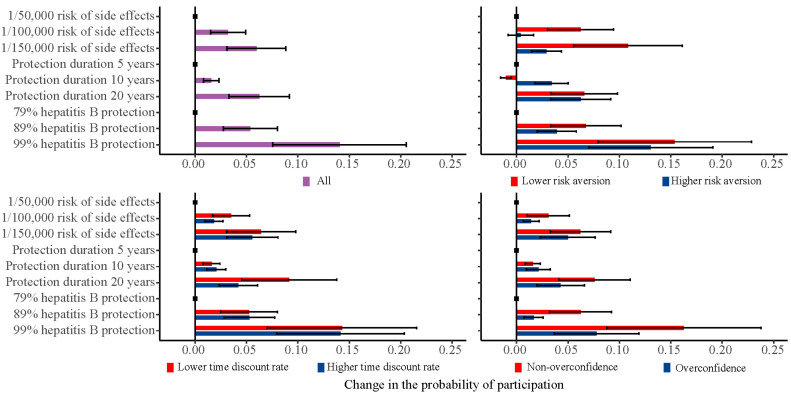
Univariate marginal estimates for predicted probability of participation.

Figure 1 shows the univariate marginal change in the probability of individuals taking a vaccination. In our total sample, changes in the protection rate from 99% or 89% to 79%, protection duration from 20 years to five years, and risk of serious side-effects from 1/150,000 to 1/50,000 had a relatively large impact on the predicted probability of individuals taking a vaccination. A decreased risk of serious side-effects from 1/50,000 to 1/150,000 had a relatively large 10.1% (95% CI: 5.3% to 15.0%) impact on the expected uptake among the low risk aversion group, but a relatively small 2.9% (95% CI: 1.4% to 4.4%) impact among the high risk aversion group. Compared to the high time discount rate group, an increased protection duration from five years to 20 years had a relatively large 8.5% (95% CI: 4.0% to 13.0%) impact on the expected uptake in the low time discount rate group. An increased protection rate from 79% to 99% had a 16.2% (95% CI: 8.7% to 23.7%) impact on the predicted probability of uptake among the non-overconfident group, but a relatively small 7.9% (95% CI: 3.8% to 12.0%) impact among the overconfident group.

## 4. Discussion

This study used discrete choice experiment modelling to elicit the preferences for HBV vaccinations, investigated the association between individual’s behavioral factors and vaccination decisions, and examined how preferences vary across different individual behavioral factor characteristics. Understanding behavioral differences in the vaccination decision and preference for a vaccine is particularly important for designing more effective policy to increase the vaccination rate. The risk of side-effects, duration of protection, and protection rate were shown to influence adults’ vaccination decisions and preferences for HBV vaccination. Individuals with lower time discount rates, non-overconfidence or high risk aversion were more likely to choose a vaccine. Low risk aversion individuals showed a higher preference for lower risk of side-effects; lower time discount rate individuals showed a higher preference for longer protection duration; and non-overconfidence individuals showed a higher preference for higher hepatitis B protection and willingness to pay more.

This study found the protection rate, duration of protection, risk of side-effects, and out-of-pocket cost had an important influence on adults’ vaccination decision which is in line with previous DCE studies on preference for the HBV vaccination [25]. Protection rate (effectiveness), duration of protection, and risk of side-effects were also shown to influence preferences for HPV, influenza and rotavirus vaccinations in previous DCE studies [32,37,38,39]. In addition, DCE studies that investigated preferences for HPV vaccination found that out-of-pocket cost influence mothers’ preferences for vaccinating daughters, which is in line with our results [37].

Our results showed that high risk aversion was positively associated with the decision to be vaccinated, which is consistent with previous studies [15]. In theory, risk aversion can affect the decision of vaccination through two opposing paths. First, the fear of being infected by the disease will lead some to vaccinate and, second, the fear of the vaccine’s side-effects will prevent others from vaccinating [16]. Since the risk of getting infected is much larger than the risk of side-effects, the impact of the perceived risk of getting infected dominates the impact of the risk of side-effects.

This study found that adults with low risk aversion showed a significantly higher preference for “lower risk of side-effects” than adults with high risk aversion, which has not been addressed in previous research. This means that low risk aversion individuals will give greater weight to the risk of side-effects than high risk aversion individuals, and have higher sensitivity to any change in side-effect risks. The dominant effect of the risk of getting infection weakens in individuals with low risk aversion, and even a minor increase (1/150,000 to 1/50,000) in risk of side-effects will have a large decrease (10.1%) in the expected uptake in those people. 

This study confirms that individuals’ time preference is associated with the vaccination decision [11,15,16], and further illustrates that preferences for “protection duration” vary across different individual time discount rates. As expected, individuals with a lower time discount rate (more future oriented) were more likely to take the vaccine, and showed a higher preference for “longer protection duration” than individuals with higher time discount rate. Since the benefits of the vaccine are reaped at some uncertain future point in time, more future oriented individuals will give a high weight to the benefits of the vaccine under the certain protection duration than lower time preference individuals [12]. Individuals with a high time discount rate will give high weight to the costs since they occur around the time of vaccination [13]. In addition, individuals with lower time preference will give more weight on longer protection duration.

The current study found that overconfidence directly affects the vaccine decision while previous research only found the indirect influence via perceived probability of infection and the severity of the disease [15,16]. After controlling for perceived risk of infection and the severity of the disease, the overconfident were still less likely to take the vaccine and selected “neither vaccine” more often. A possible explanation is that individuals with high overconfidence will overestimate their own ability, such as resistance to diseases, while simultaneously underestimating the risk of infection and the severity of the disease [40,41]. In addition, compared to overconfidence, non-overconfidence shows higher sensitivity to the vaccine protection rate and lower sensitivity to the cost. Non-overconfident respondents felt more threatened by infectious diseases [3]. Therefore, they valued the effectiveness of vaccine more than others, and cared less about the cost than others.

The results of this paper have important policy implications. Adult decision-making frequently deviates from rationality, with behavioral factors playing an important role in the decision and preference for vaccinations. Interventions by health policymakers should consider irrational aspects of human behavior and should targeted interventions to address behavioral differences. For example, vaccine interventions should target the behavioral factors that imped vaccination. For individuals with low risk aversion, their high sensitivity to adverse side-effects means that public policies should address misperceptions about vaccine side-effects. In terms of time preference, policy measures should emphasize the protection duration, which will improve the uptake among future oriented individuals; deploying incentives might be an effective intervention for individuals with higher time discount rates [3,7]. When people do not vaccinate because of overconfidence, the dissemination of information on disease risks and severity, or lowering the vaccination cost, may help raise the vaccination rate [3,15].

Although this study enhances the understanding of the behavioral factors’ differences in the preference for vaccination, it has several limitations. First, the study is only able to demonstrate adults’ preference for the attributes and levels presented [42]. While this study undertook an extensive literature review and interviews with experts in the field of HBV vaccination to guard against bias of omitted variables, certain meaningful attributes may not have been included. Second, our sample only contains information from one province of China, with a relatively small sample size, so the generalizability to populations outside of this region requires further studies undertaken in other provinces and countries. Finally, although this study analyzed how the demand and preference varies across different levels of risk aversion, time discount rate, and overconfidence, the analysis cannot demonstrate whether the preferences differ between other behavioral factors, such as altruism [43] and status quo bias [16]. These variables should be included in future studies.

## 5. Conclusions

Individuals with lower time discount rates, non-overconfidence, or high risk aversion were more likely to choose to vaccinate. Low risk aversion individuals showed a higher preference for the lower risk of side-effects, lower time discount rate individuals showed a higher preference for longer protection duration, and non-overconfidence individuals showed a higher preference for higher hepatitis B protection and willingness to pay more. Interventions should be targeted to address the behavioral determinants that imped vaccinations.

## Figures and Tables

**Table 1:** Attributes and levels.

Attributes	Levels
Risk of serious side-effects	1/50,000; 1/100,000; 1/150,000
Protection duration (years)	5; 10; 20
Protection rate against HBV (%)	79; 89; 99
Out-of-pocket cost (RMB)	0; 30; 60; 90

**Table 2:** Respondent characteristics.

Characteristics	All Sample (N = 353)
Socioeconomic factors	
Sex	
Female	164 (46.5)
Male	189 (53.5)
Age group	
Under 40	213 (60.3)
41+	140 (39.7)
Education level	
Low education	131 (37.1)
High education	222 (62.9)
RMB income group (monthly)	
≤2000	145 (41.1)
2001+	208 (58.9)
Health status	
Poor	153 (43.3)
Good	200 (56.7)
Perceived risk of HBV infection	
Low	185 (52.4)
High	168 (47.6)
Perceived severity of HBV	
Low	126 (35.7)
Moderate	92 (26.1)
High	135 (38.2)
Behavioral factors	
Risk aversion	
Higher	193 (53.82)
Lower	163 (46.18)
Time discount rate	
Higher	171 (48.44)
Lower	182 (51.56)
Overconfidence	
Yes	98 (27.76)
No	255 (72.24)

**Table 3:** Mixed logit model results for a discrete choice experiment addressing adults’ preferences for HBV vaccination.

	Model 1	Model 2
Constant	7.558(0.830) ***	6.990(1.467) ***
Attribute		
Out-of-pocket cost	−0.010(0.003) ***	−0.010(0.003) ***
Risk of side-effects (ref. = 1/50,000)		
1/100,000	0.215(0.096) *	0.226(0.097) *
1/150,000	0.418(0.097) ***	0.415(0.092) ***
Protection duration (ref. = 5 years)		
10 years	0.109(0.096)	0.110(0.095)
20 years	0.456(0.095) ***	0.439(0.092) ***
Protection rate (ref. = 79%)		
89%	0.363(0.096) ***	0.365(0.094) ***
99%	0.993(0.107) ***	0.977(0.103) ***
Socioeconomic factors		
Neither × Male		0.311(0.668)
Neither × Age 40+		−3.079(0.799) ***
Neither × High education		0.290(0.822)
Neither × Income 2000 RMB+		0.344(0.892)
Neither × Good health status		−0.235(0.511)
Neither × High risk of infection		−1.070(0.580)
Neither × Perceived moderate severity		−0.229(0.695)
Neither × Perceived high severity		−1.738(0.656) **
Behavioral factors		
Neither × Lower risk aversion		2.049(0.497) ***
Neither × Lower time discount rate		−1.185(0.594) *
Neither × Non-overconfidence degree		−1.166(0.558) *

Notes: Standard errors in parentheses. *** *p* <0.001, ** *p* <0.01, * *p* <0.05.

**Table 4:** Mixed logit model results with interactions for a discrete choice experiment addressing adults’ preferences for HBV vaccination.

	Model 3 Lower Risk Aversion	Model 4 Lower Time Discount Rate	Model 5 Non-Overconfidence
Constant	7.870 (0.940) ***	6.976 (0.915) ***	6.597 (0.684) ***
Attribute			
Out-of-pocket cost	−0.011 (0.004) **	−0.010 (0.004) **	−0.024 (0.006) ***
Risk of side-effects (ref. = 1/50,000)			
1/100,000	0.030 (0.139)	0.114 (0.118)	0.098 (0.168)
1/150,000	0.202 (0.127)	0.341 (0.118) **	0.347 (0.172) *
Protection duration (ref. = 5 years)			
10 years	0.240 (0.129)	0.127 (0.120)	0.147 (0.174)
20 years	0.443 (0.126) ***	0.258 (0.116) *	0.298 (0.168)
Protection rate (ref. = 79%)			
89%	0.267 (0.130) *	0.315 (0.121) **	0.114 (0.177)
99%	0.916 (0.142) ***	0.859 (0.133) ***	0.547 (0.184) **
Interaction terms			
Out-of-pocket cost × covariate	−0.001 (0.005)	0.000 (0.005)	0.018 (0.006) **
Risk of side-effects (ref. = 1/50,000)			
1/100,000 × covariate	0.433 (0.208) *	0.143 (0.182)	0.136 (0.209)
1/150,000 × covariate	0.598 (0.198) **	0.131 (0.192)	0.121 (0.211)
Protection duration (ref. = 5 years)			
10 years × covariate	−0.319 (0.198)	−0.009 (0.181)	−0.029 (0.211)
20 years × covariate	0.054 (0.191)	0.413 (0.176) *	0.283 (0.204)
Protection rate (ref. = 79%)			
89% × covariate	0.220 (0.197)	0.059 (0.181)	0.343 (0.213)
99% × covariate	0.218 (0.204)	0.177 (0.192)	0.669 (0.227) **

Notes: Standard errors in parentheses. *** *p* <0.001, ** *p* <0.01, * *p* <0.05.
